# Case Report: The Injectable Contraceptive as a Contributing Factor to Mental State In a Young Female With Autism Spectrum Disorder and Intellectual Disability

**DOI:** 10.1192/bjo.2025.10762

**Published:** 2025-06-20

**Authors:** Jenifer Salmons, Marianne Bergman, Alaa Martin

**Affiliations:** 1 Essex Partnership University Trust, Wickford, United Kingdom; 2 Hertfordshire Partnership Foundation Trust, Hatfield, United Kingdom

## Abstract

**Aims:** This case study explores the psychiatric and physical health complexities in a 28-year-old female service user with Autism Spectrum Disorder (ASD) and Intellectual Disability, focusing on the interplay between neuropsychiatric diagnoses, hormonal treatments, and significant mental health deterioration. It also examines the impact of hormonal changes on mood and behaviour, highlighting potential misdiagnosis of emotional instability versus neurodevelopmental conditions. The service user has a history of polycystic ovary syndrome (PCOS), irritable bowel syndrome, and Benign Rolandic Epilepsy (seizure-free since age 13). She has engaged with mental health services since adolescence, carrying multiple diagnoses including generalized anxiety disorder, post-traumatic stress disorder and emotionally unstable personality disorder (EUPD). Her mental health worsened suddenly and significantly following a switch from an oral progesterone contraceptive to the Depo-Provera injection, prompting inpatient psychiatric care.

**Methods:** A thorough medical and psychiatric evaluation was conducted during the nine-month inpatient admission. This included a mental state examination, routine blood tests, CT head imaging, and extensive collateral history collection. Medication adjustments were made including trials of SNRI and SSRI medication, and multidisciplinary therapeutic interventions were provided. Her Depo-Provera was not re-administered. Her PCOS diagnosis was confirmed and she was started on metformin. A diagnosis of ASD was implemented seven months into the admission and her EUPD diagnosis removed. Her depressive and anxious symptoms were noted to be cyclical, worsening before her menstruation. Following MDT review, she was started on an oral contraceptive with good evidence in pre-menstrual syndrome and PCOS (estradiol with nomegestrol)

**Results:** The service user presented with severe depression, anxiety, and active suicidal ideation, including multiple attempts to leave the ward to act on her plans. Initial physical and neurological workups were unremarkable. The pre-admission switch to Depo-Provera was identified as a likely contributing factor to her deterioration, as no other psychosocial triggers were found. She was subsequently detained under the Mental Health Act due to ongoing suicidality. Despite intensive psychiatric and therapeutic interventions, her mood remained persistently low – however after initiating an appropriate contraceptive, her symptoms showed some stabilization. Her risk of self-harm persisted.

**Conclusion:** This case highlights the potential influence of hormonal changes on psychiatric symptoms in women with complex neurodevelopmental disorders such as ASD. It also raises important considerations about the potential misdiagnosis of personality disorders in neurodivergent individuals and the need for careful management of hormonal treatments in this population. Further research into the hormonal impact on mood disorders in neurodivergent patients is warranted.

